# Set cover-based methods for motif selection

**DOI:** 10.1093/bioinformatics/btz697

**Published:** 2019-09-17

**Authors:** Yichao Li, Yating Liu, David Juedes, Frank Drews, Razvan Bunescu, Lonnie Welch

**Affiliations:** Department of Electrical Engineering and Computer Science, Ohio University, Athens, OH 45701, USA

## Abstract

**Motivation:**

*De novo* motif discovery algorithms find statistically over-represented sequence motifs that may function as transcription factor binding sites. Current methods often report large numbers of motifs, making it difficult to perform further analyses and experimental validation. The motif selection problem seeks to identify a minimal set of putative regulatory motifs that characterize sequences of interest (e.g. ChIP-Seq binding regions).

**Results:**

In this study, the motif selection problem is mapped to variants of the set cover problem that are solved via tabu search and by relaxed integer linear programing (RILP). The algorithms are employed to analyze 349 ChIP-Seq experiments from the ENCODE project, yielding a small number of high-quality motifs that represent putative binding sites of primary factors and cofactors. Specifically, when compared with the motifs reported by Kheradpour and Kellis, the set cover-based algorithms produced motif sets covering 35% more peaks for 11 TFs and identified 4 more putative cofactors for 6 TFs. Moreover, a systematic evaluation using nested cross-validation revealed that the RILP algorithm selected fewer motifs and was able to cover 6% more peaks and 3% fewer background regions, which reduced the error rate by 7%.

**Availability and implementation:**

The source code of the algorithms and all the datasets are available at https://github.com/YichaoOU/Set_cover_tools.

**Supplementary information:**

Supplementary data are available at *Bioinformatics* online.

## 1 Introduction

Motif discovery is a *de novo* method for mining putative transcription factor binding sites (TFBSs) from a set of related genomic regions, such as promoter regions of co-expressed genes or genomic windows that are bound by transcription factors (Das and Dai, 2007; Hu *et al.*, 2005; Landt *et al.*, 2012; Tompa *et al.*, 2005). Many methods have been developed for motif discovery, including generative algorithms (Bailey *et al.*, 1994; Pavesi *et al.*, 2004); discriminative methods (Huggins *et al.*, 2011; Smith *et al.*, 2005); deep learning approaches (Lee *et al.*, 2018; Quang and Xie, 2016); and ensemble methods (Jin *et al.*, 2009; Van Heeringen and Veenstra, 2011). Recent motif discovery tools have been optimized to handle massive ChIP-Seq datasets and to utilize ChIP-Seq specific information. For example, HMS (Hu *et al.*, 2010) uses a Bayesian model that integrates sequencing depth information. ChIPMunk (Kulakovskiy *et al.*, 2010) uses an iterative approach that incorporates peak shape information. Genome wide Event finding and Motif discovery (Guo *et al.*, 2012) is a k-mer-based method that identifies spatial binding constraints. Identification of co-enriched motifs is also an important problem when interpreting ChIP-seq peaks. One common method is to apply a co-occurrence statistical test, such as Homer-annotatepeaks (Heinz *et al.*, 2010) and MAmotif toolkit (Sun *et al.*, 2018). More advanced approaches include training a machine learning model and using feature importance to select top ranking motifs. For example, SeqUnwinder trained a multi-class logistic regression model based on k-mer frequencies using a time-series Lhx3 ChIP-seq dataset and identified Zfp281 and Oct4 as cofactors during induced motor neuron programing (Kakumanu *et al.*, 2017).

Individual motif discovery methods often fail to identify a single motif that covers all of the binding regions from a ChIP-Seq experiment (Al-Ouran *et al.*, 2018). Moreover, ensemble motif discovery methods tend to generate large numbers of motifs, which are infeasible to validate experimentally. For example, the ENCODE project (Consortium *et al.*, 2012) has produced hundreds of ChIP-seq experiments. Therefore, systematic methods for selecting motifs are needed. Kheradpour and Kellis (2014) approached this problem by: (i) manually clustering 427 ChIP-seq datasets into 84 transcription factor groups; (ii) producing an initial set of motifs using a set of five motif discovery tools; (iii) developing an enrichment method to select up to 10 motifs per transcription factor group. In our previous study, we developed a greedy set cover algorithm to address the same issues (Al-Ouran *et al.*, 2018), by finding a small number of motifs that cover all binding regions. This article introduces an enhanced version of the motif selection problem, which yields substantial improvement in solution quality by also considering background sequence coverage.

The addition of background sequence coverage, while more biologically relevant, complicates the underlying optimization problem. Ideally, we wish to (i) cover as much of the foreground as possible, (ii) cover as little of the background as possible and (iii) select the smallest set of motifs. Modeling such a multi-objective optimization problem is notoriously difficult as there can be multiple optimal solutions. For instance, the Positive Negative Partial Set Cover Problem (PNPSCP) (Miettinen, 2008) addresses (i) and (ii) by minimizing the sum of the number of foreground sequences that are not covered and the number of background sequences that are covered. Unfortunately, not addressing (iii) means that an optimal solution could contain many motifs, which again, may be infeasible to validate experimentally. In this work, we address this issue with two complementary approaches. In the first approach, we modify the PNPSCP to include the number of motifs as part of the optimization function and solve the modified PNPSCP via tabu search. In the second approach, we define a new optimization problem, namely the Minimum Discriminative Set Cover Problem (MDSCP), where the objective is to find the minimum number of motifs subject to constraints on (i) and (ii). We model this problem via linear programing and produce solutions to this problem via a randomized algorithm.

In the results, we show that set cover algorithms outperformed the enrichment method developed by Kheradpour and Kellis in terms of foreground coverage, background coverage, error rate and number of motifs. Moreover, our algorithms also identified putative cofactors for six transcription factors, including GATA and BRCA1.

In the remainder of this article, the authors formally define the new motif selection problems, solve the problems via two methods: tabu search and relaxed integer linear programing (RILP), and demonstrate the effectiveness of the solutions by analyzing ChIP-Seq data from the ENCODE project (Consortium *et al.*, 2012).

## 2 Materials and methods

Traditional motif discovery algorithms output a large number of motifs that are often infeasible to validate via laboratory experiments. The goal of the new motif selection problem is to find a small set of motifs that covers all the regions of interest while minimizing the number of false positives (i.e. covering the background sequences). In this section, we define the motif selection problem in terms of the modified PNPSCP and the MDSCP. Last, we describe our evaluation datasets and methodology.

### 2.1 Mapping the motif selection problem to a variant of the PNPSCP

A formal statement of the PNPSCP problem is as follows: given a positive set $P=\{p_{1},p_{2},\ldots ,p_{\pi }\}$, a negative set $N=\{n_{1},n_{2},\ldots ,n_{\nu }\}$ and a collection $M=\{m_{1},m_{2},\ldots ,m_{k}\}\subseteq 2^{P\cup N}$, the objective is to find a subset of *M*, denoted by $M^{*}$, such that

$$\mathrm{cost}(P,N,M^{*})=|P\setminus \underset{m\in M^{*}}{⋃}m|+|N\cap \underset{m\in M^{*}}{⋃}m|$$

is minimized (Miettinen, 2008). Note that the cost function represents the number of misclassified elements, which consists of the number of uncovered positive elements and the number of covered negative elements.

To introduce the motif selection problem, consider a motif discovery setting where a set of foreground sequences is given as $P=\{p_{1},p_{2},\ldots ,p_{\pi }\}$ and a set of background sequences is given as $N=\{n_{1},n_{2},\ldots ,n_{\nu }\}$. The output from a motif discovery algorithm or an ensemble of algortihms is a set of motifs denoted as $M=\{m_{1},m_{2},\ldots ,m_{k}\}$. Next, motif scanning is performed; motifs are mapped to the foreground and background sequences to get the information on whether a motif occurs in a sequence. A motif *m_j_* is said to cover a sequence *s_i_* if the motif *m_j_* occurs in the sequence *s_i_*. The solution to the motif selection problem is represented by a vector $\vec{x}=(x_{1},x_{2},\ldots ,x_{k})$, where

$$x_{i}=\{\begin{array}{ccc} 1 & & \text{if}\;\;m_{i}\;\;\text{is}\;\;\text{part}\;\;\text{of}\;\;\text{the}\;\;\text{solution}\\ 0 & & \text{otherwise} \end{array}$$

Let $M^{*}$ be the set of selected motifs, where $m_{i}\in M^{*}$ if *x_i_* = 1. Then the motif selection problem is to minimize the following cost function:

(1) $$\beta \times |M^{*}|+(1-\beta )\times \mathrm{Error}$$

The cost function consists of two parts: one is the number of selected motifs (i.e. $\left|M^{*}\right|$), the other one is the percentage of misclassified sequences (i.e. *Error*). It is different from the original PNPSCP formulation, thus we call it a variant of PNPSCP. β∈[0,1] is a scaling factor for the two parts, with a default value of $\frac{1}{k+1}$ (so that the ranges of the two parts are equal). The *Error* function is denoted as:

(2) $$\alpha \times \frac{\left|P\setminus {⋃}_{m\in M^{*}}m\right|}{\left|P\right|}+(1-\alpha )\times \frac{\left|N\cap {⋃}_{m\in M^{*}}m\right|}{\left|N\right|}$$

A weight factor α∈[0,1] with a default value of 0.5, is used to specify the relative importance between covering more foreground sequences and covering fewer background sequences.

#### 2.1.1 The tabu search approach

Tabu search (Glover and Laguna, 1998) is a metaheuristic local search method. It starts with a randomly generated initial solution ${\vec{x}}^{0}$ then searches the neighborhood of ${\vec{x}}^{0}$, denoted by $N({\vec{x}}^{0})$, for better solutions. The neighborhood generation function used in this study involves flipping binary values (see Gendreau, 2003).

Traditional local search methods, such as hill climbing, update current solution if they find a better solution in the neighborhood and thus result in local optima. In contrast, tabu search alleviates this issue by employing two strategies: (i) tabu search accepts non-improving moves when better moves are unavailable in the neighborhood of current solution and (ii) tabu search uses a short-term memory structure, called tabu list, to store recently visited solutions and prevent selecting solutions that are visited previously.

Algorithm 1 The tabu search algorithm for motif selection (Gendreau, 2003; Maischberger, 2011)

 ${\vec{x}}^{0}$: Initial solution

 ${\vec{x}}^{*}={\vec{x}}^{0}$: Current best solution

 *Tenure*: The size of the tabu list

 $f({\vec{x}}^{*})$: The cost of ${\vec{x}}^{*}$

 ForeCov$\left({\vec{x}}^{*}\right)$: The foreground coverage of ${\vec{x}}^{*}$

 $N({\vec{x}}^{*})$: The neighborhood of ${\vec{x}}^{*}$

 $\tilde{N}({\vec{x}}^{*})$: The ‘accessible’ subset of $N({\vec{x}}^{*})$ (i.e. non-tabu or allowed by aspiration)

 *λ*: The foreground coverage incremental threshold

 **while**¬terminate()**do**

  Update_flag = FALSE

  **for**$\vec{x}\prime \in \tilde{N}({\vec{x}}^{*})$**do**

   **if**$(|\vec{x}\prime |>|{\vec{x}}^{*}|)\wedge \mathrm{ForeCov}(\vec{x}\prime )-\mathrm{ForeCov}({\vec{x}}^{*})<\lambda$**then**

    pass

   **else**

    **if**$f(\vec{x}\prime )<f({\vec{x}}^{*})$**then**

     ${\vec{x}}^{*}=\vec{x}\prime$

     Update_flag = TRUE

    **end if**

   **end if**

  **end for**

  **if** Update_flag is TRUE **then**

   Delete the oldest entry if the tabu size >Tenure

   Add ${\vec{x}}^{*}$ to the tabu list

  **else**

  **end if**

 **end while**

 **return**${\vec{x}}^{*}$

Because the tabu list may prohibit reaching better solutions (if intermediate moves to such solutions are tabu; Gendreau, 2003), it may be necessary to revoke tabus (i.e. allow one visited solution to be non-tabu). Such operations are called aspiration criteria. We employ the ‘best so far’ aspiration criterion, which allows moving to a neighborhood solution if its objective value is close to the current best solution (Gendreau, 2003; Maischberger, 2011).

#### 2.1.2 The tabu search algorithm

The METSlib framework is used to implement the tabu search algorithm. METSlib (Maischberger, 2011) is a metaheuristic modeling framework and optimization toolkit based on the programing language C++. Algorithm 1 shows the pseudocode for the tabu search algorithm. It starts with an initial solution ${\vec{x}}^{0}$. The initial solution is a set of all motifs. ${\vec{x}}^{*}$ is the current best solution. f() calculates the cost value defined in Equation (1). ForeCov() calculates the percentage of covered foreground sequences for a given solution. *λ* is used as a foreground coverage incremental threshold, meaning that the best solution is replaced by the current solution only if it adds *λ*% or more foreground coverage. The similar threshold is used in the greedy set cover algorithm (Al-Ouran *et al.*, 2018). $N({\vec{x}}^{*})$ is a set of *k* neighborhood solutions of ${\vec{x}}^{*}$, which are generated by flipping the binary value at each position of ${\vec{x}}^{*}$. $\tilde{N}({\vec{x}}^{*})\subset N({\vec{x}}^{*})$ consists of two parts: (i) non-tabu neighborhood solutions and (ii) tabu solutions that are allowed by aspiration. $\tilde{N}({\vec{x}}^{*})$ should be updated after enumerating all neighborhood solutions of ${\vec{x}}^{*}$.

Our implementation uses the following termination criteria: (i) mets::noimprove_termination_criteria. If the total number of non-improving iterations exceeds a maximum number, then the tabu search is stopped. (ii) mets::threshold_termination_criteria. This termination criterion terminates the tabu search when the cost reaches a certain threshold. The tabu list uses mets::simple_tabu_list. The aspiration criterion uses mets::best_ever_criteria. In each iteration, we search for $\vec{x}\prime$ in the neighborhood of ${\vec{x}}^{*}$ that minimizes the cost function. If the cost of $\vec{x}\prime$ is less than the current best solution and its incremental foreground coverage is greater or equal to *λ*, then the best solution is assigned to $\vec{x}\prime$. Otherwise, the non-improving counter adds 1.

The tabu search algorithm runs in iterations. In each iteration, it takes O(|M|*(|P|+|N|)) time to calculate the cost function. Since the neighborhood of the current solution contains at most |M| solutions, it can take at most $O(|M{|}^{2}*(|P|+|N|))$ steps to finish every iteration. Thus, the tabu search algorithm given above has a time complexity of $O(\max*|M{|}^{2}*(|P|+|N|))$, where *max* denotes the total number of iterations.

### 2.2 Mapping the motif selection problem to MDSCP

Unlike the tabu approach, which tries to minimize the number of motifs and the number of misclassified sequences at the same time, in this section, we introduce a parameterized version of the motif selection problem, which we refer to as the MDSCP.

Definition 2.1 MDSCP: Given a foreground set *P*, a background set *N*, a set *M* containing subsets of P∪N and integers *k* and *j*, find a subset $M^{*}\subseteq M$ of minimum cardinality satisfying the following two constraints:

1. $$|\underset{m\in M^{*}}{\cup }m\cap P|\geq |P|-k,$$
  *i.e.* *at most k elements in P are* ***not*** *covered by some set in* $M^{*}$, and
2. $$|\underset{m\in M^{*}}{\cup }m\cap N|\leq j,$$
  *i.e.* *at most j elements of N are covered by the sets in* $M^{*}$.

MDSCP is shown to be NP-complete by reducing the set cover problem to it (i.e. set k=0,j=|N|). Therefore, finding exact and fast algorithms for MDSCP is difficult. However, we can use standard techniques to bound the optimal value of the MDSCP.

#### 2.2.1 Integer linear programing characterizations

In this section, we present a 0−1 integer linear programing characterization of MDSCP and explore how to use this for approximation.

Definition 2.2 Given an instance 〈P,N,M,k,j〉 of MDSCP, we define the following 0−1 linear programing variant of this instance. Let m=|M|+|P|+|N|. Let $\vec{x}$ be a 0–1 vector of size m such that $\vec{x}=\vec{u}\vec{v}\vec{w}$, where $\vec{u}$ has size $|M|,\;\vec{v}$ has size |P| and $\vec{w}$ has size |N|. The objective is to find a 0−1 vector $\vec{x}$ such that the following linear constraints are satisfied and the number of 1’s in $\vec{u}$ is minimized.

1. *For every element i of P*,
  $$\sum_{i\in M_{j}}u_{j}-v_{i}\geq 0.$$
  *Notice that, since both* $u_{j}\in \{0,1\}$*and* $v_{i}\in \{0,1\}$*, then if v_i_ = 1, there must be at least one u_j_ = 1 such that* $i\in M_{j}$.
2. *For every element i of P, let* $K_{i}=|\{M_{j}|i\in M_{j}\}|$*, and let*
  $$\sum_{i\in M_{j}}u_{j}-K_{i}*v_{i}\leq 0.$$
  *Notice that, since both* $u_{j}\in \{0,1\}$*and* $v_{i}\in \{0,1\}$*, then if v_i_ = 0, there is no u_j_ where u_j_ = 1 and* $i\in M_{j}$*. This guarantees that, if v_i_ = 0, then i in P is not covered.*
3. *For every element i of N, let*
  $$\sum_{i\in M_{j}}u_{j}-w_{i}\geq 0.$$
  *Notice that, since both* $u_{j}\in \{0,1\}$*and* $w_{i}\in \{0,1\}$*, then if w_i_ = 1, there must be at least one u_j_ = 1 such that* $i\in M_{j}$.
4. *For every element i of N, let* $K_{i}=|\{M_{j}|i\in M_{j}\}|$*, and let*
  $$\sum_{i\in M_{j}}u_{j}-K_{i}*w_{i}\leq 0.$$
  *Notice that, since both* $u_{j}\in \{0,1\}$*and* $w_{i}\in \{0,1\}$*, then if w_i_ = 0, there is no u_j_ where u_j_ = 1 and* $i\in M_{j}$*. This guarantees that, if w_i_ = 0, then i in N is not covered.*
5. $$\sum_{i=1}^{\left|P\right|}v_{i}\geq |P|-k,$$
  *i.e.* *at least all but k of the foreground elements are covered.*
6. $$\sum_{i=1}^{\left|N\right|}w_{i}\leq j,$$
  *i.e.* *at most j of the background elements are covered*.

We refer to this instance as *MDSCP_ILP_*. We note that the optimal solution to the integer linear programing formulation *MDSCP_ILP_* is equivalent to the optimal solution to MDSCP. However, both problems are NP-complete. Fortunately, the integer linear programing formulation provides a natural avenue for approximation via relaxation. In this case, the relaxed version of *MDSCP_ILP_* is the linear program where the constraints that $x_{i}\in \{0,1\}$ are replaced by $x_{i}\in [0,1]$. We refer to the relaxed problem as *MDSCP_LP_*.

#### 2.2.2 The RILP algorithm

The RILP algorithm (Algorithm 2) contains two steps. The first step is to obtain an optimal solution ${\vec{x}}^{*}$ to the *MDSCP_LP_* problem (which can be computed via GNU Linear Programming Kit; Makhorin, 2008). The second step is to solve the *MDSCP_ILP_* problem through a randomized algorithm.

If the randomized algorithm halts, it is clear that the solution $M^{*}$ covers at least |P|−k elements of *P*. However, it is possible that the given solution covers more than *j* elements of *N*. In this situation, there are two possible approaches: (i) consider this solution a failure, and (ii) consider this a solution that satisfies only one of the two constraints. Our software uses approach (ii).

The RILP algorithm takes at most O(max*|M|*(|P|+|N|)) steps to complete, notwithstanding the cost of computing the optimal solution to *MDSCP_LP_* via linear programing, given that the sets $M^{*}$, *P_M_* and *N_M_* are implemented via bit-vectors and each set *m_i_* is implemented via a balanced binary tree.

Algorithm 2 The RILP algorithm for motif selection

 Compute ${\vec{x}}^{*}$, the optimal solution to *MDSCP_LP_*.

 $M^{*}=\emptyset$; $P_{M}=\emptyset$; $N_{M}=\emptyset$.

 *iter* = 0

 **while** not done and *iter* < *max* **do**

  iter=iter+1

  **for** each set *m_i_* **do**

   add *m_i_* to $M^{*}$ with probability $u_{i}^{*}$.

   **if** *m_i_* is added to $M^{*}$then

    $P_{M}=P_{M}\cup (m_{i}\cap P)$.

    $N_{M}=N_{M}\cup (m_{i}\cap N)$.

   **end if**

  **end for**

  if $|P_{M}|\geq |P|-k$, halt and return $M^{*}$.

 **end while**

### 2.3 Evaluation methodology

To evaluate our methods, we used the ChIP-Seq datasets and the predicted binding motifs from (Kheradpour and Kellis, 2014). The authors analyzed 427 ChIP-Seq experiments and grouped them into 84 transcription factor groups based on homology. Ensemble motif discovery was done using five existing motif discovery methods: MEME (Bailey *et al.*, 1994), AlignACE (Hughes *et al.*, 2000), Trawler (Ettwiller *et al.*, 2007), MDscan (Liu *et al.*, 2002) and Weeder (Pavesi *et al.*, 2004). The top 10 most enriched motifs for each factor group were reported. The enrichment score was computed based on the fraction of motif instances in the bound regions (as detected by ChIP-seq).

Three set cover-based methods were evaluated against the enrichment method (Kheradpour and Kellis, 2014), including a greedy set cover algorithm (Al-Ouran *et al.*, 2018) and the aforementioned tabu search and RILP methods. The greedy set cover algorithm uses the ‘maximum uncovered-first’ rule (Al-Ouran *et al.*, 2018). Therefore, a motif will be added to the set until all the sequences are covered. This method doesn’t consider background sequences.

Our methods are validated using 55 factor group datasets because the known motifs of these factors are available; each of the datasets contains pooled regions (q-value ≤ 0.01) across all the ChIP-Seq experiments of the given factor. To generate evaluation datasets, 10 000 random peaks were selected per factor group dataset. A few numbers of datasets, including SIX5, ATF3, ZEB1, PBX3, MXI1, ZBTB33, NR2C2, BHLHE40, ZBTB7A, BRCA1, POU5F1, NFE2, PRDM1, HSF and SREBP contained <10 000 peaks, so all the peaks were used. The same number of randomly selected background regions from Kheradpour and Kellis (2014) was added to the evaluation datasets. In other words, the evaluation datasets contain a balanced number of foreground sequences and background sequences.

**Fig. 1.**
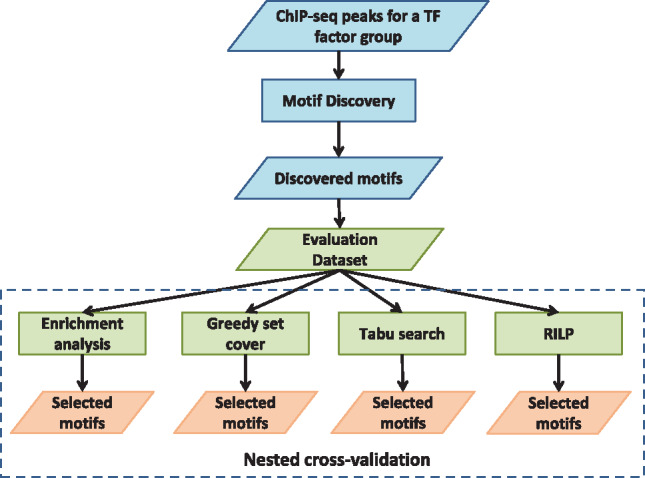
Motif selection evaluation pipeline using ENCODE datasets. The blue boxes represent the motif discovery steps in (Kheradpour and Kellis, 2014). The discovered motifs were obtained from Kheradpour and Kellis (2014). All the ChIP-Seq datasets from the same transcription factor group (defined in Kheradpour and Kellis, 2014) were combined and duplicate peaks were removed. The evaluation datasets contain 10 000 random selected peaks, 10 000 random selected background sequences and the discovered motifs. Two new motif selection algorithms (i.e. the tabu search algorithm and the RILP algorithm), the greedy algorithm (Al-Ouran *et al.*, 2018), and the enrichment method (Kheradpour and Kellis, 2014) were evaluated using nested CV

Figure 1 shows the pipeline used for evaluating the motif selection methods. The sets of all discovered motifs for each factor group were adopted from (Kheradpour and Kellis, 2014). The evaluation datasets contain foreground sequences (i.e. bound regions), background sequences and the corresponding motifs discovered in that factor group. Motif scanning was done using find individual motif occurrences (FIMO) with default parameters (e.g. *P*-value cutoff = 1e−4) (Grant *et al.*, 2011). In a recent study of motif scanning tools (Jayaram *et al.*, 2016), FIMO was the top performer comparing to Matrix-Scan (part of the RSAT suite) (Turatsinze *et al.*, 2008), Clover (Frith *et al.*, 2004), Patser (Turatsinze *et al.*, 2008) and PossumSearch (Beckstette *et al.*, 2006). Since a motif can either occur or not occur in a sequence [i.e. zero or one occurrence per sequence, the ZOOP model (Bailey *et al.*, 1994)], it is natural to produce a boolean matrix to represent the occurrence information, where each row is a sequence and each column is a motif. Together with the class label (i.e. foreground sequence or background sequence), it is the input to the enrichment method and the motif selection methods. The optimization process is to find the best combination of columns (i.e. combination of motifs) in terms of the number of uncovered foreground sequences, the number of covered background sequences and the number of selected motifs. The evaluation procedure used a nested cross-validation (CV) approach (see Supplementary Fig. S1; Chen *et al.*, 2008). Nested CV can reduce the bias and give a better estimation of the error than the traditional CV methods (Varma and Simon, 2006). For the Greedy method, filter_level was searched from 1 to 20%. For the tabu search method, tenure (i.e. controlling the tabu list size) was set to be 0.2, 0.4 or 0.6 and delta (i.e. incremental coverage cutoff, same as filter_level in the Greedy method) was set to be 2%. For the RILP method, maximal uncovered foreground percent and maximal covered background percent were searched from 10 to 50%. The evaluation program was run at the Ohio Supercomputer Center. Each algorithm for each dataset was run for 100 h with 8 cores and 64G memory. The nested CV program ran in parallel. Due to excessive memory usage, the tabu search algorithm did not finish four datasets: AP1, CTCF, MYC and TATA (which contain 244, 853, 372 and 248 motifs, respectively).

The motif selection methods were evaluated using the following metrics:

1. **Foreground coverage (ForeCov):** The fraction of foreground sequences that contain the selected motifs. The algorithms attempt to maximize this metric.
2. **Background coverage (BackCov):** The fraction of background sequences that contain the selected motifs. The algorithms attempt to minimize this metric.
3. **Error rate:** The fraction of uncovered foreground sequences (i.e. False negatives) and covered background sequences (i.e. False positives).
4. **Number of motifs:** The number of selected motifs returned by motif selection algorithms. This number should be minimized.

Individual motifs were evaluated based on a Fisher exact test (Lin *et al.*, 2015) where the 2 × 2 contingency table was created with the following values: (i) the number of foreground sequences with at least one occurrence of a given motif; (ii) the number of foreground sequences with no occurrence of the given motif; (iii) the number of background sequences with at least one occurrence of the given motif; (iv) the number of background sequences with no occurrence of the given motif.

## 3 Results and discussion

Using the set cover-based methods, we are able to identify a small set of motifs for each TF group with high foreground coverage and low background coverage. This section provides a comparison of the results obtained by the set cover methods and the enrichment method (Kheradpour and Kellis, 2014). Additionally, we discuss biological insights provided by the motifs identified by the set cover methods.

### 3.1 Comparison of set cover-based methods

Three set cover algorithms were evaluated on the same 55 TF group datasets used by the enrichment method (Kheradpour and Kellis, 2014). Unlike the enrichment method, which calculates an enrichment score for each motif and then selects the top 10 motifs, the set cover methods iteratively optimize a group of selected motifs.

**Fig. 2.**
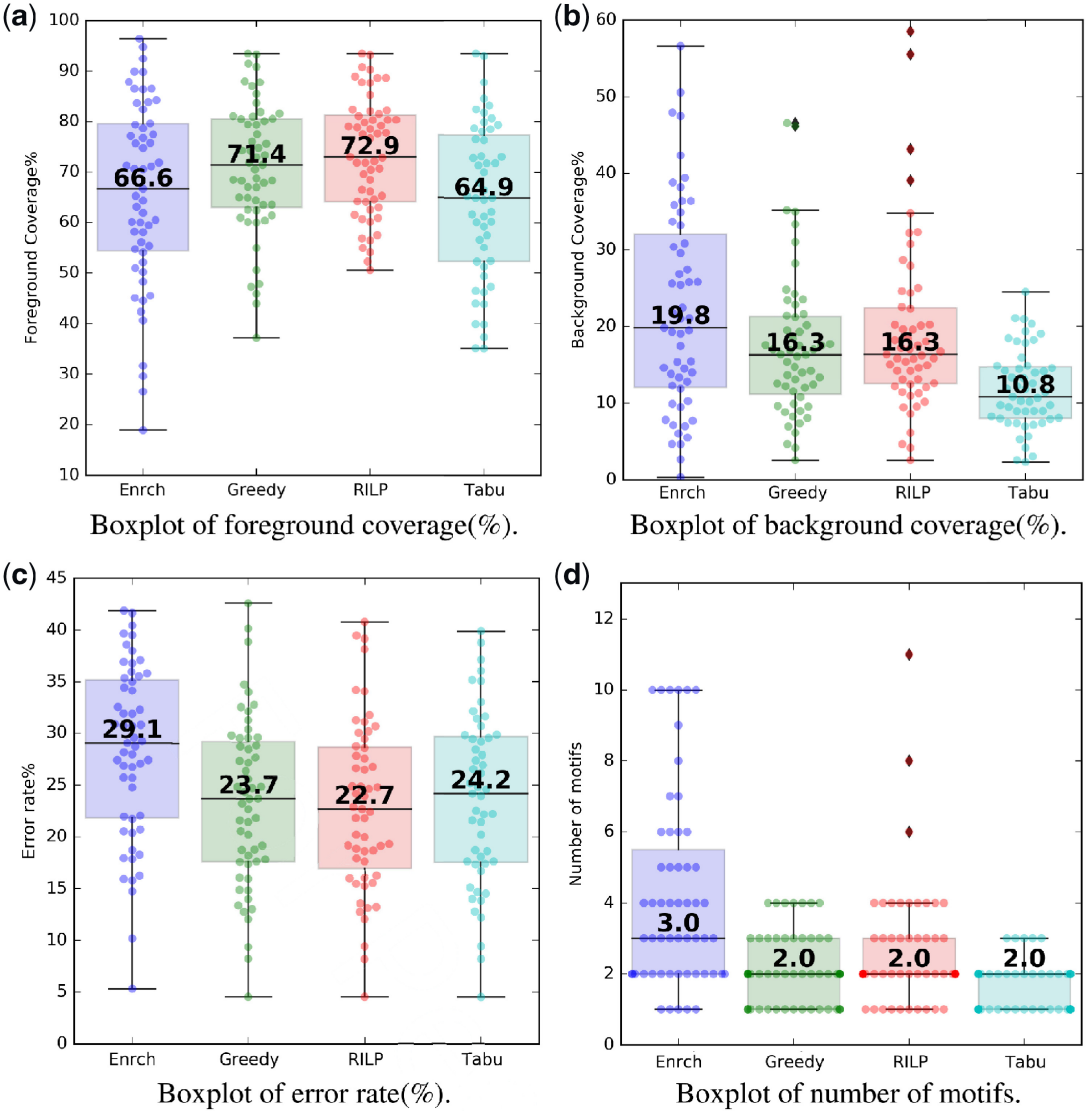
Boxplots of the four evaluation metrics. Median values and all the data points are shown. Each data point represents the dataset of a transcription factor group. Enrch: the enrichment method (Kheradpour and Kellis, 2014). Greedy: the greedy algorithm for motif selection (Al-Ouran *et al.*, 2018). RILP: the RILP algorithm for motif selection. Tabu: the tabu search algorithm for motif selection

The foreground coverage represents the fraction of ChIP-Seq regions that contain the selected motifs. As shown in Figure 2a, the median foreground coverage of the enrichment method is 66.6%, even though it is 1.7% higher than the tabu search method, it is 4.8 and 6.3% lower than the greedy method and the RILP method, respectively. Specifically, the enrichment method failed to cover more foreground sequences in 41 TF groups (see Supplementary Fig. S2a), suggesting that simply selecting the top motifs based on a sequence enrichment method can fail to account for all sequences of interest. With respect to the foreground coverage metric, the RILP method performed the best.

The background coverage shows the fraction of randomly selected regions not identified by ChIP-Seq that contain the selected motifs. In other words, it represents the false positive rate (because the motifs are not expected to occur in the background sequences). As shown in Figure 2b, the median background coverage of the enrichment method is 19.8%, which is 3.5% higher than the greedy method and the RILP method, respectively. With respect to the background coverage metric, the tabu search method performed the best.

The error rate represents the percentage of misclassified sequences if the selected set of motifs is used to predict the regions bound by a TF. The median error rate of the enrichment method is 29.1% (Fig. 2c). All three set cover-based methods have a lower median cost (than the enrichment method) and the RILP method has the lowest median cost of 22.7%.

The median number of selected motifs doesn’t vary much (i.e. two or three motifs) for these methods (Fig. 2d). However, their ranges can differ significantly. For example, the enrichment method has a range from 1 to 10 and the RILP method has a range from 1 to 12. On the other hand, the greedy and tabu search methods pick only one to four motifs for each TF group. It is worth noting that the RILP method selected one to four motifs in most cases (52/55) (see Supplementary Fig. S2b). Therefore, the set cover-based methods select fewer motifs than the enrichment method and the tabu search method generally picks the smallest number of motifs.

Our results demonstrate the effectiveness of set cover approaches in solving the sequence coverage problem (Al-Ouran *et al.*, 2018). For example, the enrichment method produced the highest foreground coverage in NRF1, CTCF, REST, SPI1 and ETS (Supplementary Fig. S2a). However, in all the aforementioned five TF groups, the enrichment method reported a larger number of motifs (Supplementary Fig. S2b); it selected 10 CTCF motifs while the set cover methods selected only 1 motif. The number of discovered motifs is greatly reduced using set cover-based methods. The minimal description length principle favors hypotheses that describe the biological data using fewer symbols than needed (Grunwald, 2004). In this vein, the set cover methods discover few motifs, which in turn tend to cover few background sequences (Supplementary Fig. S2c) and thus produce low-cost solutions (Supplementary Fig. S2d). Overall, when compared with the enrichment method, the RILP algorithm selected two motifs (median number) and was able to cover 6% more peaks and 3% fewer background regions, which reduced the error rate by 7%.

### 3.2 Shared motifs between the solutions of set cover-based methods and the enrichment method

The three set cover-based methods have found the same motifs in seven factor groups as reported in (Kheradpour and Kellis, 2014). As shown in Table 1, these shared motifs occur more frequently in the bound regions than in the background regions. For example, TFAP2_disc2 occurs in 76.3% of the TFAP2 binding peaks and yet only 9.7% of the background sequences. TAL1_disc1 matches the binding motif of GATA. It has been shown that TAL1 acts as a cofactor for GATA3 (Ono *et al.*, 1998). More recently, Moreau *et al.* (2016) has identified ‘GATA1, FLI1 and TAL1 as a minimal and sufficient combination of TFs to induce the formation of MK precursors from hPSCs’, which is relevant to transfusion medicine. PBX3_disc2 matches the known MEIS1 motif (Kheradpour and Kellis, 2014), which is consistent with the known cooperative binding activity of PBX3 and MEIS1 (Bischof *et al.*, 1998). Interestingly, it is known that PBX3 and MEIS1 work cooperatively in hematopoietic cells to drive acute myeloid leukemia (AML) (Li *et al.*, 2016), suggesting PBX3_disc2 might play an important role in the progression of AML. Our results show that the set cover-based methods were able to re-identify enriched motifs as reported by the enrichment method.

**Table 1.** Shared motifs between the three set cover-based methods and the enrichment method

Motif name	Motif Logo	ForeCov	BackCov
TFAP2_disc2	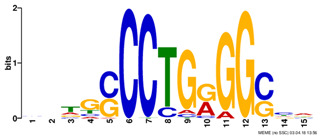	76.3%	9.7%
POU5F1_disc1	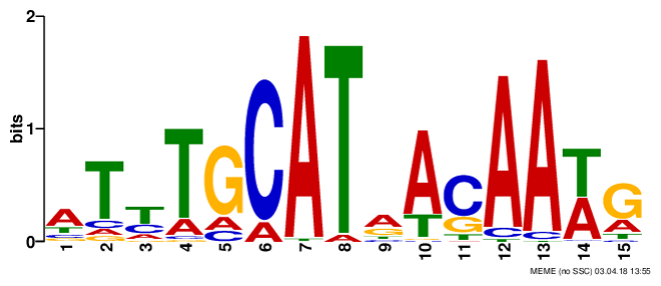	71.8%	12.2%
REST_disc3	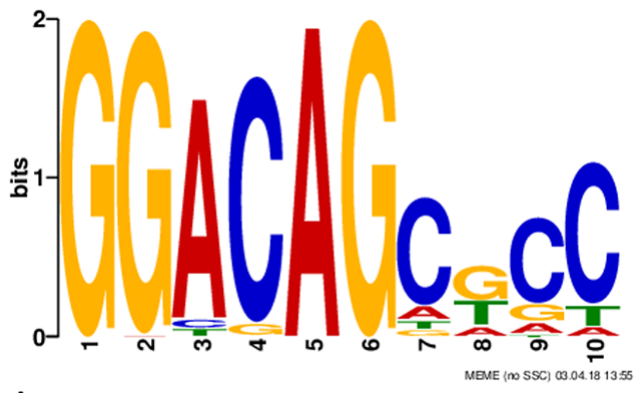	60.7%	8.4%
TAL1_disc1	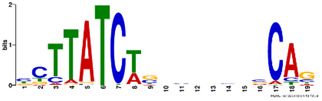	47.3%	7.0%
ZNF143_disc3	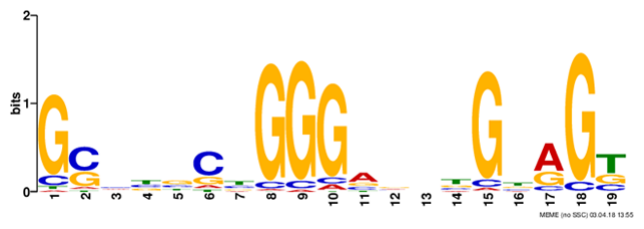	39.8%	11.9%
PAX5_disc1	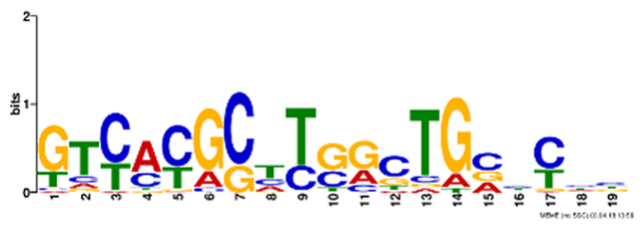	37.8%	5.6%
PBX3_disc2	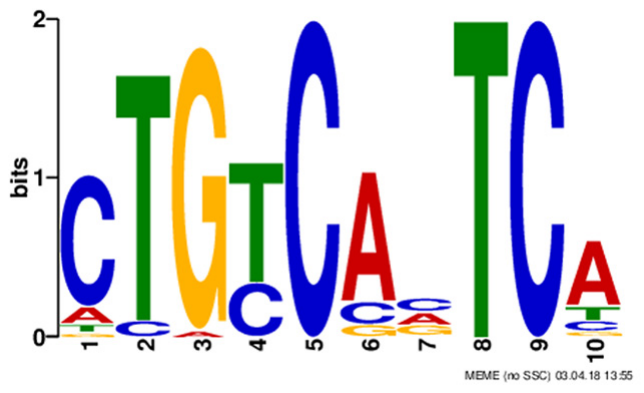	37.3%	7.4%

*Note: Motif names used in this table are adopted from Kheradpour and Kellis (2014).*

### 3.3 Putative cofactors identified by set cover-based methods

To explore whether the set cover-based methods identified any known motifs that were missed by the enrichment method (Kheradpour and Kellis, 2014), we took the union of motifs selected by the set cover methods and filtered out the motifs that were similar to the enrichment discovered motifs. The remaining motifs were matched to 579 JASPAR 2018 vertebrates non-redundant motifs (Khan *et al.*, 2018) using TOMTOM (Gupta *et al.*, 2007) with q-value cutoff at 0.01, resulting in six motifs (Table 2). A Fisher exact test (Lin *et al.*, 2015) showed that these motifs were significantly enriched in the ChIP-Seq peaks. Interestingly, three motifs in HEY1, GATA and EP300 factor groups all matched the binding motif of ZNF263. It has been reported that HEY1 and ZNF263 are highly expressed (fold change ≥ 25) in the CD34^+^ cell line (Gomes *et al.*, 2002), suggesting that they might be cofactors. The ZBTB33 motif found in the BRCA1-bound regions is consistent with the finding that BRCA1 might ‘bind ZBTB33 to perform their functions in DNA repair and genome maintenance’ (Wang *et al.*, 2012). Moreover, both BRCA1 and ZBTB33 are strongly associated with TP53 (Szklarczyk *et al.*, 2015), suggesting they might have a cooperative function in cancer. RXRA and RXRG are retinoic acid receptor RXR-alpha and RXR-gamma, respectively. Hence, it is expected to see the binding motif of RXRG that we observed in RXRA bound regions. In summary, the motifs identified by the set cover methods provide new potential insights regarding the genomic biology of gene regulation.

**Table 2.** Putative cofactors discovered by the three set cover-based methods

Factor group	Discovery tool	Motif logo	ForeCov	BackCov	Fisher *P*-value	JASPAR match	TOMTOM *P*-value
HEY1	MEME	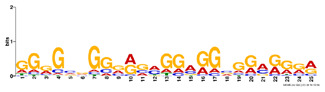	67.6%	22.0%	0	MA0528.1 (ZNF263)	3.1E-13
BRCA1	AlignACE	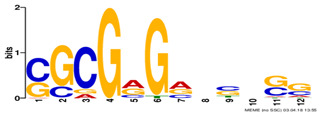	46.8%	2.8%	0	MA0527.1 (ZBTB33)	4.0E-06
PBX3	AlignACE	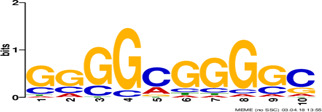	31.8%	8.3%	7.8E-281	MA0516.1 (SP2)	3.2E-08
RXRA	MEME	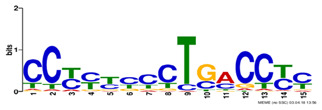	39.1%	22.8%	2.7E-137	MA1149.1 (RXRG)	1.8E-11
GATA	MEME	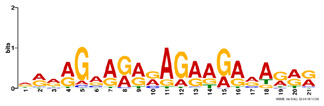	40.8%	34.9%	1.6E-17	MA0528.1 (ZNF263)	6.0E-08
EP300	MEME	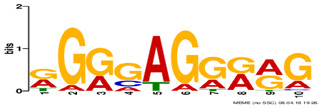	30.1%	25.0%	1.0E-15	MA0528.1 (ZNF263)	9.2E-09

*Note:* These six motifs were matched to known TFBSs and were not reported by the enrichment method (Kheradpour and Kellis, 2014). The significance of motif enrichment (i.e. Fisher *P*-value) in the bound regions versus background sequences was calculated based on a Fisher exact test (Lin *et al.*, 2015). The top known motif matches based on TOMTOM (Gupta *et al.*, 2007) from the JASPAR (Khan *et al.*, 2018) database are shown.

### 3.4 Improved motif results by the set cover-based methods

The results show that the set cover algorithms improve the motif set discovered in ENCODE ChIP-seq experiments. Specifically, the set cover methods increased the foreground coverage by at least 35% for 11 TF groups (see Supplementary Fig. S2a). The methods also discovered motifs for POU2F2 (a key regulator for B cells and neuronal cells; Latchman, 1996) and BRCA1 (a well-known tumor suppressor). The set cover methods decreased the error rate by at least 10% for 9 TF groups (see Supplementary Fig. S2c), including BRCA1 and MXI1 (an oncogenic transcription factor). Given the improvement in foreground coverage and the decrease in error rate, the set cover-based methods have produced an improved, high-quality motif analysis result for ChIP-seq data.

## 4 Conclusion

Current motif discovery tools often produce a large number of DNA motifs, making it difficult to gain biological insight or to perform experimental validation. One way to select fewer motifs is to perform an enrichment analysis; this type of analysis evaluates individual motifs and outputs a motif list (e.g. ranked by enrichment score). Users can set their own threshold and select the top motifs. In contrast, the motif selection problem provides a way to find a concise set of key regulatory motifs that maximizes foreground coverage and minimizes background coverage. Specifically, the motif selection algorithms do not explicitly evaluate individual motifs; they look for a set of motifs by performing a combinatorial optimization.

This article contributes two new set cover-based methods to solve the motif selection problem. Tabu search is an effective metaheuristic method that uses adaptive memory programing to explore the solution space in a manner that avoids repetitively searching in the region of a local optimum. This method performed the best in terms of background coverage and number of motifs. RILP is a classic method for solving set cover problems. The relaxed constraints guarantee that the algorithm finds optimal solutions in the linear space. Then it uses a randomized algorithm to pick the motifs based on probabilities returned by the optimal solution. This method performed the best in terms of foreground coverage and error rate, and it also selected one to four motifs in most cases. In terms of time complexity, both the tabu search and the RILP method are linear with respect to the number of input sequences. The number of motifs (i.e. —M—); however, is different between the two methods. It is still linear for the RILP method, but it is quadratic for the tabu search method, which means that for inputs with large number of motifs, the RILP method is more efficient. Taken together, the RILP method is recommended as the single algorithm of choice, because it provides a small set of motifs that covers most of the foreground sequences and few of the background sequences. Another good approach is to select the set of motifs identified by one or more of the set cover-based algorithms.

Identification of putative cofactor binding sites is important for biological interpretation of ChIP-seq peaks. It is worth noting that the analysis of the set cover-based methods showed that they not only rediscovered motifs that were reported by the enrichment method but also identified known motifs representing putative cofactors that were missed by the enrichment method. In summary, the set cover-based methods improved ChIP-seq motif content significantly, including >35% increment in foreground coverage for 11 TFs. When applying a nested CV framework and comparing to the motifs reported by Kheradpour and Kellis, the RILP algorithm selected fewer motifs and was able to cover 6% more peaks, 3% fewer background regions and 7% lower error rate. New biological insights were gained from the four new putative cofactors that were missed by the enrichment method.

Future work may include expansion of the set cover algorithms to include a multi-cover approach, which is based on the set multi-cover problem (Chekuri *et al.*, 2009). For example, it is known that CTCF binds to a 33/34 bp region that consists of the CTCF motif and a shorter secondary motif (i.e. M2). With the multi-cover constraint, each CTCF peak is required to be covered by at least two different motifs.

## Funding

L.W. was funded by the Graduate Education and Research Board Program of Ohio University.

*Conflict of Interest*: none declared.

## Supplementary Material

btz697_Supplementary_Data
