# Supplementary material for: Set cover-based methods for motif selection
Source: Bioinformatics. 2019 Sep 17;36(4):1044–51. doi: 10.1093/bioinformatics/btz697 (PMC7703758; doi:10.1093/bioinformatics/btz697)
Supplement: btz697_Supplementary_Data [file btz697_supplementary_data.pdf]

Supplemental Figures: Set Cover Based Methods for Motif Selection in ChIP-Seq Data

Yichao Li, Yating Liu, David Juedes, Frank Drews, Razvan Bunescu, Lonnie Welch

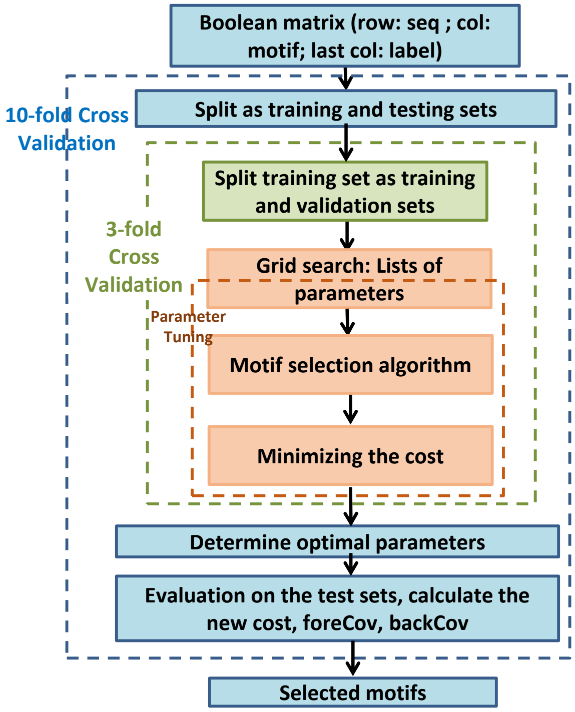

Fig. S1: Nested cross-validation workflow for evaluating motif selection algorithms. A boolean matrix containing the motif occurrence information was given as input. Then the data was split in an outer CV and an inner CV. Inside each inner CV, a grid search of the parameters was done. Final optimal parameters were obtained in the outer CV by selecting the most frequent optimal parameters in the inner CV. Lastly, the final selected motifs were obtained using the whole dataset and the optimal parameters.

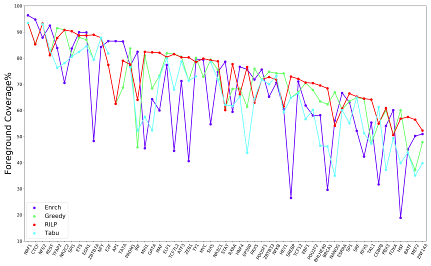

(a) Line plot of foreground coverage(%).

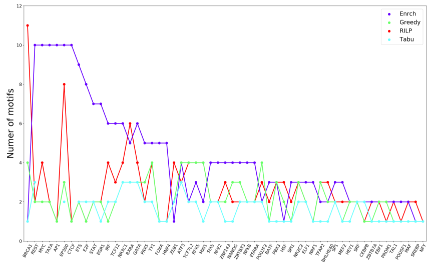

(b) Line plot of number of motifs.

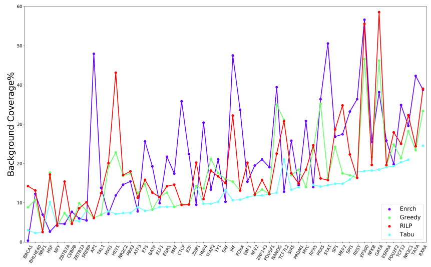

(c) Line plot of background coverage(%).

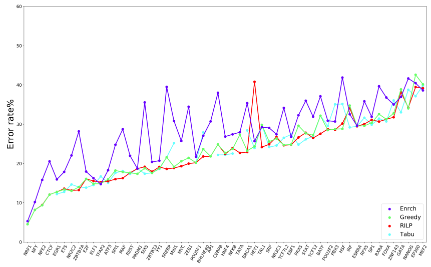

(d) Line plot of error rate(%).

Fig. S2: Line plots of the 4 evaluation metrics. The horizontal axis shows the 55 transcription factor groups. Enrch: the enrichment method. Greedy: the greedy algorithm for motif selection. RILP: the RILP algorithm for motif selection. Tabu: the tabu search algorithm for motif selection.
